# Unexpected negative-pressure pulmonary edema after tracheostomy: two case reports

**DOI:** 10.1186/s40981-025-00777-w

**Published:** 2025-02-27

**Authors:** Taichi Kotani, Yusuke Naito, Chie Okuda, Shota Sonobe, Junji Egawa, Masahiko Kawaguchi

**Affiliations:** Department of Anesthesiology, Nara Medical University, Kashihara, Japan

**Keywords:** Negative-pressure pulmonary edema, Tracheotomy, Mechanical airway obstruction, Functional airway obstruction, Withdrawal syndrome

## Abstract

**Background:**

Negative-pressure pulmonary edema (NPPE) often develops with upper airway obstruction, and is uncommon in secured airways, for example, after tracheostomy. Herein, we report two cases of NPPE post-tracheostomy.

**Case presentation:**

Case 1: A 69-year-old man underwent prophylactic tracheotomy for possible airway obstruction secondary to glottic carcinoma. Two hours after awakening from general anesthesia, he had difficulty expectorating and developed NPPE due to airway secretions obstructing the tracheostomy tube.

Case 2: An 11-year-old boy was admitted to the intensive care unit for continuous hemodiafiltration on a ventilator under sedation. On the 76th day, the day after the tracheostomy was performed, the patient developed patient-ventilator asynchrony due to sedative withdrawal syndrome. The postulated primary mechanism was functional airway obstruction due to patient-ventilator asynchrony.

**Conclusion:**

These cases highlight the need to consider NPPE, even in patients with an airway secured with a tracheostomy.

## Background

Negative-pressure pulmonary edema (NPPE) is a non-cardiogenic pulmonary edema caused by a sudden decrease in intrathoracic pressure during upper airway obstruction. It involves considerable inspiratory pressure in the trachea, caused by upper airway obstruction, which decreases intrathoracic pressure and increases pulmonary capillary pressure [1]. Treatment for NPPE involves relieving airway obstruction by securing the airway, such as by tracheal intubation [2]. However, mechanical ventilation does not guarantee successful NPPE treatment. Improper management of mechanical ventilation may lead to the onset of NPPE. Herein, we describe two cases of NPPE despite tracheostomy.

## Case presentation

### Case 1

**Fig. 1:**
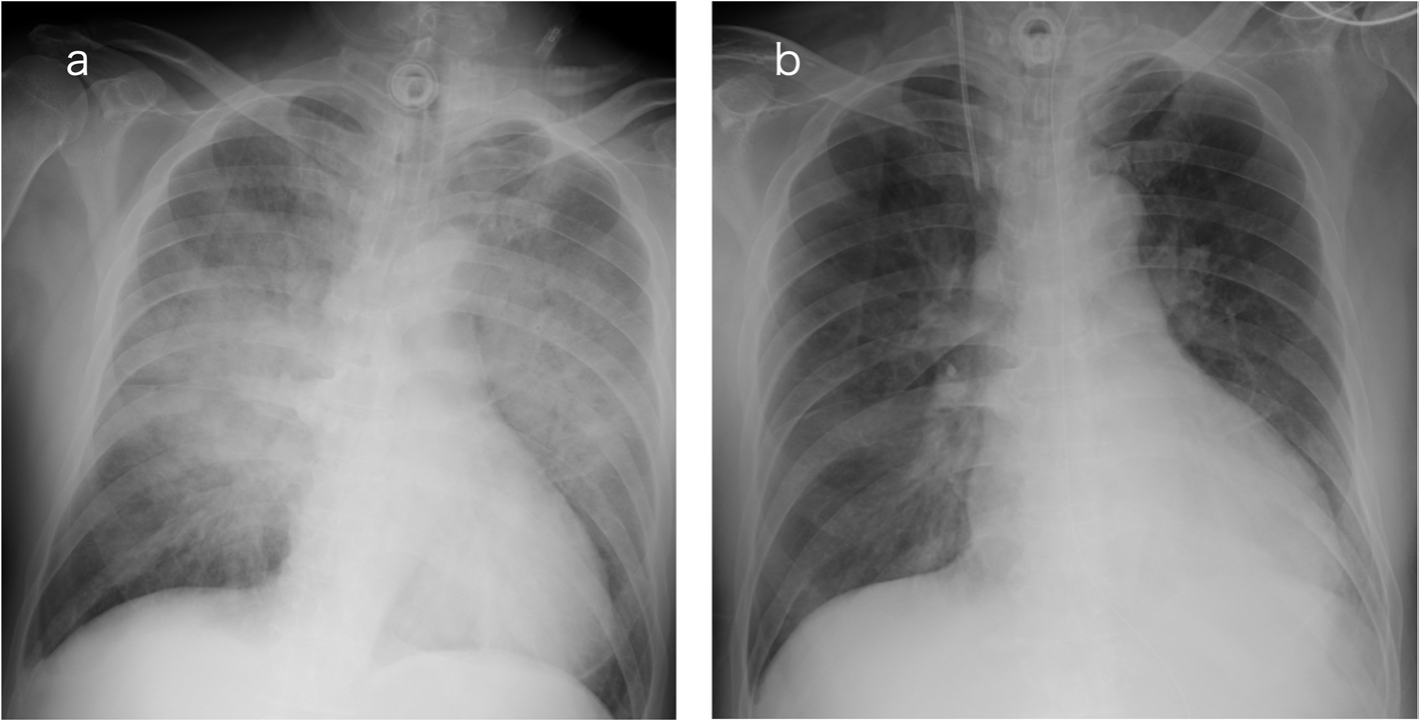
Chest radiography images of Case 1. **a** At the onset of pulmonary edema after tracheostomy. **b** Forty-eight hours after onset and treatment of pulmonary edema

A 69-year-old man (height, 174 cm; weight, 79 kg) underwent prophylactic tracheostomy under general anesthesia for possible glottal stenosis secondary to glottal cancer. After general anesthesia, the patient was spontaneously breathing and returned to the ward, receiving oxygen at 5 L/min. After 2 h, he had difficulty expectorating and developed increased inspiratory effort associated with systemic hypertension (173/133 mmHg), tachycardia (140 beats/min), foamy sputum production, and oxygen desaturation to 80%. The patient was admitted to the intensive care unit (ICU) for positive-pressure ventilation. Immediately after admission, sputum was suctioned from the tracheostomy tube, and the tube lumen was observed to be almost occluded by the stiff sputum, which was then aspirated using a broncho-fiberscope, and the patient was placed on a ventilator. He responded well to positive-pressure ventilation, and the PaO_2_/FiO_2_ ratio improved from 67 to 200 without diuretics or other medications. Chest radiography revealed bilateral infiltrative shadows (Fig. 1a). Echocardiography showed no signs of left heart failure, supporting a diagnosis of NPPE. Two days later, the radiographic infiltrative shadows improved (Fig. 1b). The patient was weaned off ventilation and was transferred to the ward after confirmation of expectoration ability.

### Case 2

**Fig. 2:**
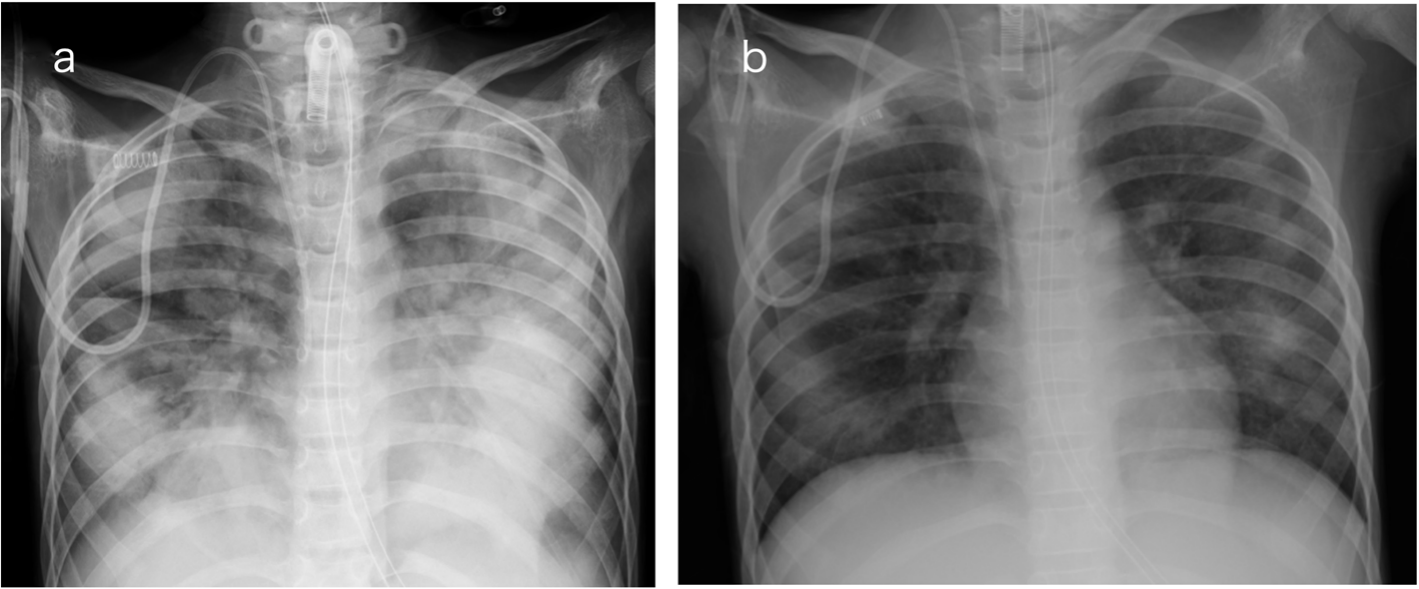
Chest radiography images of Case 2. **a** After tracheostomy with pulmonary edema. **b** Forty-eight hours after onset and treatment of pulmonary edema

An 11-year-old boy (height, 137 cm; weight, 22.9 kg) diagnosed with acute lymphoblastic leukemia developed acute kidney injury. Continuous hemodiafiltration (CHDF) was required, but difficulties in maintaining bed rest for extended periods were expected because of a history of attention-deficit hyperactivity disorder since the age of 8 years. The patient was admitted to the ICU for deep sedation, tracheal intubation, and ventilation. Fentanyl, dexmedetomidine, and midazolam were used for sedation, and CHDF was performed. As tolerance to sedative medications developed, especially to midazolam, ketamine and morphine were added. On the 57th day, the patient was weaned from CHDF, and the sedatives were tapered in preparation for extubation. However, the development of withdrawal syndrome made extubation impossible, and on the 74th day, a tracheostomy became necessary. The patient was transferred to the ward on the following day, still receiving dexmedetomidine. Two days later, he developed breathing difficulties. His respiratory rate was 70 breaths/min with oxygen desaturation to 60%. The patient was placed on a ventilator with settings including pressure support ventilation (PSV) pressure of 18 cmH_2_O and positive end-expiratory pressure of 6 cmH_2_O. However, the patient’s breathing was asynchronous with the ventilator, and he was readmitted to the ICU because the PaO_2_/FiO_2_ ratio decreased to 40. A small amount of foamy secretion, not sufficiently stiff to obstruct the lumen, was aspirated from the tracheostomy tube. Chest radiographs showed bilateral infiltrative shadows (Fig. 2a); however, echocardiography ruled out the diagnosis of left heart failure. We suspected the recurrence of withdrawal syndrome and re-sedated the patient, switched to positive-pressure ventilation, and added diuretics. The patient responded well, and the PaO_2_/FiO_2_ ratio improved significantly to 350 on the following day, leading to the diagnosis of NPPE. The radiographic infiltrative shadows improved (Fig. 2b). Subsequently, he was transferred back to the ward on the ventilator while receiving dexmedetomidine and other sedatives, such as clonidine, triclofos sodium, and diazepam. Withdrawal syndrome did not recur. He was eventually weaned off positive-pressure ventilation and underwent tracheostomy closure.

## Discussion

We encountered two cases of NPPE after tracheostomy. Because NPPE usually develops with upper airway obstruction, it rarely develops in a secured airway such as that after a tracheostomy. NPPE develops when the increased inspiratory effort associated with upper airway obstruction decreases the intrathoracic pressure. A sudden drop in intrathoracic pressure creates a pressure differential within the blood vessels and alveoli, where the fluid moves from the pulmonary veins to the pulmonary interstitium, suggesting that NPPE is caused by hydrostatic pressure differences between pulmonary capillaries and interstitium rather than increased permeability of the pulmonary capillaries [1]. Lower edema fluid/plasma protein ratio reported in patients with NPPE than those with high-permeability edema also supports this hypothesis [3–5].

The diagnostic criteria for NPPE include (1) upper airway obstruction; (2) sudden onset of respiratory difficulty, hypoxia, and hypercapnia; (3) presence of frothy sputum; and (4) chest radiographic findings showing diffusely increased density, widened vascular shadows, and bilateral, central alveolar, and interstitial infiltrates [6]. Although our cases lack mechanical upper airway obstruction as commonly observed after extubation, they suggest that similar conditions can occur both mechanically and functionally, even in patients with secured airways.

In Case 1, sputum caused mechanical airway obstruction of the tracheostomy tube. On the other hand, Case 2 had difficulty breathing and developed tachypnea due to withdrawal syndrome, although secretions in the trachea unlikely caused airway obstruction. Ineffective ventilator triggering occurs when the next inspiratory effort begins before the expiratory outflow is completed under positive-pressure ventilation [7]. In this case, the high PSV pressure setting caused hyperinflation, which, combined with high airway resistance (due to tracheostomy airway), resulted in a prolonged expiratory time. As a result, the next inspiratory effort was initiated before the expiration was completed. Still, it did not reach the flow trigger sensitivity, leading to an ineffective triggering, which caused the negative intrathoracic pressure. Tachypnea and high PSV pressure may have caused ineffective triggering, resulting in intense negative endotracheal pressure. With residual expiratory flow, strong inspiratory effort is required to meet the ventilator flow-triggered air delivery criteria. NPPE can occur due to functional airway obstruction, even without mechanical occlusion. Although rare, a similar case of NPPE after tracheostomy has been reported [8]. They noted that acute or chronic obstruction resolves after tracheotomy can lead to severe dyspnea with NPPE, as seen in Case 2. Furthermore, a pediatric study reported that children have higher thoracic compliance, which allows decreased airway pressure to greatly affect intrathoracic pressure [9]. They observed that NPPE developed in 7% of children with upper airway obstruction. This is higher than the recently reported 3% incidence of NPPE in adults with upper airway obstruction [10].

The most crucial treatments for NPPE include relieving the upper airway obstruction and administering positive-pressure ventilation [2]. Several studies have reported that NPPE resolves within 24–48 h in most cases [1, 2, 11]. Because NPPE results from hydrostatic rather than increased permeability edema, it responds well to positive-pressure ventilation and improves rapidly. The two cases we encountered illustrated this point. If pulmonary edema does not improve with these treatments, the differential diagnoses should include acute heart failure, acute respiratory distress syndrome, aspiration pneumonia, or pulmonary thromboembolism [12].

In conclusion, we encountered two cases of NPPE after tracheostomy due to mechanical and functional airway obstruction. These cases demonstrate that NPPE should be considered even in patients with an airway secured with a tracheostomy.

## Data Availability

Not applicable.

## Abbreviations

NPPE: Negative-pressure pulmonary edema
ICU: Intensive care unit
CHDF: Continuous hemodiafiltration
PSV: Pressure support ventilation
